# The Emerging Role of Butyrate in Immune Tolerance

**DOI:** 10.1155/jimr/6636669

**Published:** 2026-10-27

**Authors:** Juzhong Zhang, Shuyan Shen, Jiamin Cao, Yunping Li, Boding Tong, Yuxian Shi, Yangbo Zhou, Xin Qi

**Affiliations:** ^1^ Department of Ophthalmology The Second Xiangya Hospital Central South University Changsha Hunan China csu.edu.cn; ^2^ Department of Ophthalmology The Third Xiangya Hospital Central South University Changsha Hunan China csu.edu.cn; ^3^ Department of Neurosurgery The Second Xiangya Hospital Central South University Changsha Hunan China csu.edu.cn

**Keywords:** antigen-presenting cells, butyrate, immunosuppression, lymphocyte, neutrophils

## Abstract

The intestine is the largest peripheral lymphoid organ in humans and interacts with a vast intestinal microbiota. There is a growing awareness that the intestinal microbiota plays a crucial role in training, functioning, and inducing the host immune system, affecting the overall health of the host. However, the composition of the intestinal microbiota varies with factors such as ethnicity, geographical location, and diet, making it difficult to form a universal pattern. Microbial metabolites are regarded as messengers of the intestinal microbiota, capable of generating unique molecules that humans cannot produce. Many cells of the immune system also express receptors for these molecules, playing a key role in guiding the differentiation of immune cells. Short‐chain fatty acids (SCFAs) produced through the fermentation of dietary fiber, particularly butyrate, serve as key mediators linking gut microbial metabolism to host immune regulation. Accumulating evidence indicates that butyrate contributes to the maintenance of immune homeostasis by modulating immune cell metabolism, differentiation, cytokine production, and intercellular communication. The immunological effects of butyrate cannot be simply categorized as immunosuppressive or tolerance‐inducing. Instead, its functional outcomes are shaped by multiple factors, including immune cell type, local concentration, tissue microenvironment, route of administration, and disease context. This context‐dependent nature highlights the complexity of butyrate‐mediated immune regulation and underscores the need for a more comprehensive understanding of its mechanisms of action. This review summarizes the effects of butyrate on macrophages, dendritic cells (DCs), lymphocytes, and neutrophils with respect to cellular metabolism, differentiation, cytokine secretion, and intercellular communication. In addition, common underlying mechanisms, including histone deacetylase (HDAC) inhibition, metabolic reprogramming, and G protein–coupled receptor (GPCR) signaling, together with current controversies and knowledge gaps, are discussed to provide new insights into the role of butyrate in maintaining immune homeostasis.

## 1. Introduction

The human microbiota comprises bacteria, fungi, viruses, and other microorganisms. A 70‐kg individual harbors ~3.8 × 10^13^ bacterial cells, comparable to the total number of human cells, with the gut microbiota constituting a significant proportion of this microbial population [1]. The emergence of germ‐free animals has further revealed the critical role of gut microbiota in promoting immune system development and maintaining immune homeostasis [2]. For instance, intestinal immune cells are reduced in germ‐free animal models, Paneth cells are smaller, and Peyer’s patches exhibit diminished size, demonstrating the intricate interactions between host immune cells and the gut microbiota [2]. Microbial metabolites play a significant role in signaling between host immunity and the gut microbiota. Dietary fats, proteins, fiber, and other components undergo microbial fermentation in the colon, producing short‐chain fatty acids (SCFAs), secondary bile acids, vitamin B12, and other metabolites that support nutritional, physiological, and immune homeostasis [3]. Among these metabolites, SCFAs have attracted significant attention for their critical role in immune regulation and intestinal homeostasis maintenance by inhibiting proinflammatory cytokine production and regulating immune cell differentiation [4].

SCFAs are produced through gut microbial fermentation of undigested dietary carbohydrates—particularly resistant starch and dietary fiber—with dietary and endogenous proteins contributing to a lesser extent; these metabolites primarily comprise acetate, propionate, and butyrate [5]. Substantial evidence demonstrates that SCFAs not only exert multifaceted effects on host physiology locally in the gut but also mediate systemic impacts on distal organs through gut–organ axes such as the gut–liver, gut–brain, and gut–lung axes [6–9], ameliorating diabetic nephropathy through the gut–kidney axis [10] and promoting liver regeneration through the gut–liver axis [11]. Among SCFAs, butyrate displays the broadest range of biological functions, and *Clostridium* species within the human gut microbiota serve as primary butyrate producers [6, 12].

Aberrant immune responses underlie the pathogenesis of numerous disorders, including rheumatoid arthritis, multiple sclerosis (MS), inflammatory bowel disease (IBD), and diabetes mellitus [13]. Research demonstrates that various immune‐related diseases are associated with alterations in the gut microbiota and its metabolites, while butyrate has been shown to modulate inflammatory responses and immune cell functions in several animal models and in vitro studies. The underlying mechanisms involve suppression of proinflammatory cytokine production, promotion of tolerogenic immune cell differentiation, and inhibition of immune cell activation processes, essential for maintaining immune tolerance and preventing aberrant autoimmunity. Given the exponential growth of research in this field, a comprehensive understanding of the underlying mechanisms is urgently needed to facilitate the identification of new research directions. Accordingly, this review not only summarizes the established effects of butyrate on major immune cell populations but also highlights the shared molecular mechanisms, context‐dependent effects, and unresolved controversies that shape our current understanding of its role in immune tolerance. By integrating these diverse findings, we aim to establish a coherent conceptual framework to guide future investigations and to inform the development of butyrate‐based therapeutic strategies for autoimmune diseases.

## 2. Methods

For this narrative review, we searched the PubMed database for literature published between January 1, 2005, and October 2025. We focused on studies addressing butyrate, immune cells (including T cells, B cells, macrophages, and others), and butyrate‐based treatment strategies. Keywords included (“T cell” OR “B cell” OR “Macrophage” OR “Dendritic cell” OR “NK cell” OR “Neutrophil”) AND (“butyrate”). Only English‐language original research articles and reviews were included. Studies that did not directly investigate butyrate or the interaction between butyrate and immune cells were excluded. This review aims to summarize the existing evidence by research topic, focusing on systematically organizing the current state of knowledge rather than conducting a quantitative meta‐analysis.

## 3. Effects of Butyrate on Antigen‐Presenting Cells

### 3.1. Effects of Butyrate on Macrophages

As a gut microbial metabolite, butyrate plays an important role in immune tolerance by modulating macrophage metabolism. Experimental studies have demonstrated the existence of various macrophage phenotypes in vivo, including M1‐like, M2‐like, and hybrid phenotypes [14]. However, current research focuses primarily on the M1 and M2 phenotypes. Therefore, this review will concentrate on these two phenotypes: M1 (proinflammatory phenotype), which mediates inflammatory responses, and M2 (prorepair phenotype), which promotes wound healing and produces abundant anti‐inflammatory cytokines and growth factors [15].

#### 3.1.1. Effects of Butyrate on Morphology and Function of Macrophage

Butyrate profoundly regulates both the morphology and the function of macrophages. Macrophage activation and polarization are typically accompanied by characteristic morphological changes, with M1 macrophages exhibiting a large, flattened “pancake‐like” morphology, whereas M2 macrophages display a spindle‐shaped or elongated morphology [16]. Upon lipopolysaccharide (LPS) stimulation, butyrate effectively inhibits the transition of macrophages toward the characteristic M1 phenotype, with the majority of cells retaining a spindle‐shaped or elongated M2‐like morphology [16]. M2 macrophages possess potent phagocytic capacity, enabling the efficient clearance of necrotic cells and cellular debris. This process of engulfing apoptotic cells, known as efferocytosis, activates anti‐inflammatory signaling pathways and hence promotes the resolution of proinflammatory responses [17]. Following efferocytosis, M2 macrophages increase the production of interleukin (IL)‐10, transforming growth factor‐β (TGF‐β), IL‐4, and IL‐13, which further promote efferocytosis and upregulate mannose receptor (MR) expression, thereby establishing a positive feedback loop that contributes to inflammation resolution [17].

At the molecular level, the nonreceptor tyrosine kinase (Src) and its downstream substrate focal adhesion kinase (FAK) have been shown to be induced and activated during LPS‐mediated macrophage mobilization [18]. Butyrate suppresses the transcription of both inducible nitric oxide synthase (*iNOS*) and *Src*, thus reducing their expression as well as the phosphorylation of Src and FAK [18]. In a murine model of nonalcoholic steatohepatitis (NASH), mice with sodium butyrate (NaBu) oral gavage markedly reduced macrophage recruitment to the liver, decreased their phagocytic capacity toward GFP‐labeled *Escherichia coli*, induced the expression of cleaved caspase‐3 (CC3), and was accompanied by reduced tumor necrosis factor‐α (TNF‐α) expression and fewer F4/80‐positive macrophages, indicating extensive apoptosis of LPS‐stimulated macrophages [19].

Notably, the regulatory effects of butyrate on macrophages are not unidirectional. In a hamster model of leptospirosis, oral administration of butyrate functions as an HDAC3 inhibitor and promotes reactive oxygen species (ROS) generation through monocarboxylate transporter (MCT), as a result, enhancing the bactericidal activity of macrophages rather than simply suppressing immune responses [7]. In addition, butyrate enhances the expression of hypoxia‐inducible factor‐1 (HIF‐1) by inhibiting histone deacetylase (HDAC) activity, thereby increasing the production of antimicrobial effectors and strengthening the microbial clearance capacity of macrophages [20]. Consistent with this mechanism, oral administration of a high‐inulin diet effectively alleviated *Staphylococcus aureus*–induced mastitis in mice through the production of microbiota‐derived butyrate [21].

With respect to tissue repair, butyrate‐treated M2 bone marrow–derived macrophages (M2‐BMDMs) exhibit an enhanced capacity to promote cell migration and wound healing [22]. Consistent with these findings, butyrate‐treated M2‐BMDMs maintain elevated expression of chemokine genes, including CCL2, CCL17, and CCL22 [22]. However, whether these changes directly contribute to the enhanced wound‐healing phenotype remains to be determined.

#### 3.1.2. The Effect of Butyrate on Differentiation of Macrophage

Butyrate exerts a profound influence on macrophage polarization. Studies have shown that NaBu dose‐dependently suppresses the LPS‐induced polarization of RAW264. 7 macrophages toward the M1 phenotype, consequently increasing the proportion of M2 macrophages [22, 23]. Interestingly, treatment of unpolarized macrophages (M0) with NaBu does not induce the expression of M2‐associated markers, including arginase‐1 (*Arg1*), *Fizz1*, *Ym1*, and the MR, suggesting that butyrate alone is insufficient to directly drive M2 polarization [22]. Instead, its immunomodulatory effects appear to depend largely on the inflammatory microenvironment. Under inflammatory conditions, butyrate increases the proportion of CD206^+^ macrophages, enhances *Arg1* expression, and promotes the secretion of the anti‐inflammatory cytokines IL‐4, IL‐10, and IL‐13 while simultaneously reducing *iNOS* expression and the production of proinflammatory mediators, including TNF‐α, IL‐1β, and IL‐12 [15]. Evidence from a rat model of preeclampsia further demonstrated that microbiota‐derived butyrate markedly reversed the LPS‐induced shift in the M1/M2 macrophage balance [24]. This effect was accompanied by reduced P62 protein levels and an increased LC3‐II/LC3‐I ratio, indicating that butyrate may promote macrophage autophagy [24].

The effects of butyrate on macrophage polarization are mediated primarily through the following signaling pathways.

(1) HDAC inhibition and the signal transducer and activator of transcription 6 (STAT6)/signal transducer and activator of transcription 1 (STAT1) signaling pathways: As a HDAC inhibitor, NaBu plays a pivotal role in promoting M2 macrophage polarization. IL‐4 induces the polarization of M0 toward the M2 phenotype through the activation of the STAT6 signaling pathway [22]. Experimental evidence indicates that NaBu suppresses HDAC1 expression, leading to increased histone H3K9 acetylation and subsequent activation of STAT6 phosphorylation [22]. As a consequence, the expression of M2‐associated genes, including *Arg1*—which shows the most pronounced induction—*Fizz1*, and *Ym1*, is markedly enhanced, ultimately favoring M2 polarization [22]. NaBu also exerts anti‐inflammatory effects by limiting M1 macrophage polarization. HDAC3 strongly restricts CREB‐binding protein (CBP)–mediated acetylation of STAT1, thereby facilitating STAT1 signaling [25]. In a methicillin‐resistant *S. aureus* (MRSA) pneumonia model, oral gavage of NaBu pretreatment disrupted the interaction between STAT1 and HDAC3, resulting in enhanced STAT1 acetylation and attenuation of STAT1‐driven inflammatory signaling [25]. These changes suppressed M1 macrophage polarization while promoting a shift toward the M2 phenotype. Collectively, these findings identify HDAC3 as a central molecular target through which butyrate regulates the antimicrobial function of macrophages.

NLR family pyrin domain containing 3 (NLRP3) inflammasome pathway: The NLRP3 inflammasome is a key regulator of macrophage polarization. Butyrate markedly suppresses inflammasome activation by reducing the expression of core NLRP3 inflammasome components, including NLRP3, apoptosis‐associated speck‐like protein (ASC) containing a CARD, and cleaved caspase‐1, in macrophages [26]. Conversely, NLRP3 overexpression markedly increases the expression of M1‐associated markers, including Fpr2, CD38, and GPR18, while suppressing the expression of M2‐associated markers such as CD206, CD204, and CD163, although the molecular basis underlying these changes remains unclear [26]. In the sepsis model, inhibition of the NLRP3 inflammasome by butyrate favors M2 macrophage polarization and alleviates barrier dysfunction [27]. ROS are essential mediators of NLRP3 inflammasome activation. NaBu increases the expression of superoxide dismutase (SOD) while reducing intracellular ROS and malondialdehyde (MDA) levels, creating an intracellular environment that is less permissive for NLRP3 inflammasome activation [28]. A comparable effect was observed in oxidized low‐density lipoprotein (ox‐LDL)–stimulated human monocytic leukemia cell line (THP‐1) cells, where NaBu reduced ROS production and decreased the expression of both NLRP3 and caspase‐1, ultimately limiting inflammasome activation [29]. In addition, NLRP3 functions downstream of pattern‐recognition receptor signaling and is required for the full activation of the nuclear factor kappa‐light‐chain‐enhancer of activated B cells (NF‐κB) pathway [28]. By preventing NLRP3 inflammasome activation, NaBu suppresses phosphoinositide 3‐kinase/protein kinase B (PI3K/Akt)/NF‐κB signaling and attenuates autophagy [28]. Consistent with this mechanism, treatment with NaBu markedly reduced the phosphorylation of PI3K and Akt and limited the nuclear translocation of NF‐κB p65 in advanced glycation end product (AGE)–stimulated THP‐1 cells, leading to the inhibition of AGE‐induced autophagy [28].

Other receptor‐mediated pathways: Butyrate also regulates macrophage polarization through additional receptor‐dependent pathways. Activation of peroxisome proliferator–activated receptor‐α (PPAR‐α) prevents LPS‐induced M1‐like polarization in a ligand‐dependent manner [30]. Mechanistically, activated PPAR‐α interacts with the NF‐κB subunit p65, preventing its binding to NF‐κB response elements and consequently restraining M1 macrophage polarization [30]. Computational analyses have also suggested that NaBu promotes M2 polarization while suppressing M1 polarization through the G protein–coupled bile acid receptor 1 (TGR5)/β‐arrestin 2 signaling axis, although the underlying molecular mechanism remains to be clarified [31]. As a ligand for G protein–coupled receptors (GPCRs), butyrate likewise participates in the regulation of M2 macrophages through GPCR‐dependent signaling. Evidence from LPS‐challenged G protein–coupled receptor 109AG (GPR109a)–deficient (GPR109a^−^) mice showed that loss of GPR109a did not alter the proportion of total macrophages (CD11b^+^F4/80^+^) within the CD45^+^ cell population but significantly increased the frequency of M1 macrophages while reducing the M2 population [32]. This phenotype may result from reduced suppressor of cytokine signaling 3 (SOCS3) expression accompanied by enhanced STAT1 phosphorylation, which collectively favors macrophage polarization toward the M1 phenotype [32].

Taken together, current evidence indicates that HDAC inhibition represents the principal mechanism by which butyrate regulates macrophage polarization, whereas the activation of PPAR‐α and GPCR signaling provides complementary regulatory inputs. The relative contribution of each pathway appears to depend on the cellular context and pathological microenvironment.

#### 3.1.3. Effects of Butyrate on Metabolism of Macrophage

The metabolic state of macrophages is closely linked to their functional polarization. Butyrate suppresses glycolytic metabolism by inhibiting the mammalian target of rapamycin (mTOR) pathway, shifting cellular metabolism toward oxidative phosphorylation (OXPHOS) and favoring the acquisition of an M2 phenotype [19, 27]. Unexpectedly, butyrate‐treated macrophages exhibit significantly lower ATP levels and lactate production, together with reductions in both basal and maximal respiratory capacity, indicating that OXPHOS is also compromised and that overall cellular activity is further suppressed [19] (Table 1). These observations suggest that the metabolic effects of butyrate cannot be explained simply by a shift from glycolysis to OXPHOS. Nevertheless, metabolic reprogramming induced by butyrate does not invariably result in functional suppression. During mycobacterial infection, NaBu enhances the pentose phosphate pathway by upregulating glucose‐6‐phosphate dehydrogenase (G6PDH), providing reducing equivalents for nicotinamide adenine dinucleotide phosphate (NADPH) oxidase 2 (NOX2)–dependent ROS production [36]. The resulting increase in ROS promotes macrophage apoptosis and facilitates the elimination of intracellular pathogens. A similar phenomenon has been observed during pulmonary fungal infection, where butyrate augments macrophage phagocytic activity and ROS production, improving host defense against opportunistic pathogens such as *Aspergillus* spp. [27]. These findings highlight another facet of butyrate biology, namely, its ability to elicit protective immune responses through metabolic reprogramming in the context of infection.

**Table 1:** The role of butyrate in metabolic reactions in immune tolerance cells.

Immune cell	Material	Metabolic reactions	Outcomes	Description	References
Macrophage	C57BL/6 mice	OXPHOS↑	Anti‐inflammatory M2 phenotype	Inhibition of the mTOR reduces glycolysis in macrophages and promotes a shift in metabolism toward oxidative phosphorylation, thereby inducing the M2 phenotype	[19]
Dendritic cell	C57BL/6 mice	Glycolysis↓	imDC	Downregulation of Hif1‐α expression reduces the expression of hexokinase, which is a key rate‐limiting enzyme in glycolysis, thereby inhibiting glycolysis in dendritic cells and ultimately suppressing DC maturation	[33]
T cell	Wild‐type male C57BL/6 mice	OXPHOS↑ Glycolysis↓	Tregs	Butyrate enhances OXPHOS in a PPAR‐γ–dependent manner and reduces the expression of the HIF‐1α signaling pathway to inhibit glycolysis. Both factors collectively promote Treg cell differentiation	[34]
B cell	Human peripheral blood, C57BL/6 WT mice, and DBA/1JWT mice	Tryptophan metabolism↑	Breg cell	Butyrate promotes tryptophan metabolism, enhances the synthesis of 5‐HIAA in Breg cells, supports Breg cells function via AhR, and inhibits the differentiation of GC B cell and plasmablast differentiation	[35]

Abbreviations: 5‐HIAA, 5‐hydroxyindoleacetic acid; AhR, aryl hydrocarbon receptor; Breg cell, regulatory B cell; GC B cell, germinal center B cell; imDC, immature dendritic cell; mTOR, mammalian target of rapamycin; OXPHOS, oxidative phosphorylation; PPAR‐γ, peroxisome proliferator–activated receptor‐γ; Tregs, regulatory T cells.

#### 3.1.4. Effects of Butyrate on Anti‐Inflammation of Macrophage

Promotion of anti‐inflammatory cytokine production represents another key mechanism by which butyrate establishes immune tolerance in macrophages. At the transcriptional level, butyrate suppresses stimulus‐induced expression of *IL1B*, *IL6*, *TNF*, *NLRP3*, *CD80*, and *CASP1* while upregulating the transcription of *IL4*, *IL10*, *TGFB*, and *PPARG*, accompanied by reduced inflammasome activation [19, 23, 28, 29]. These transcriptional changes are reflected at the protein level, where butyrate reduces the production of multiple proinflammatory mediators, including IL‐1β, IL‐6, TNF‐α, iNOS, CXCL9, and CCL11, while increasing the secretion of anti‐inflammatory factors such as IL‐10, IL‐13, and CX3CL1 [19, 25, 28, 29, 32, 37, 38] (Table 2). NaBu also dose‐dependently suppresses the LPS‐induced expression of the exosomal markers CD63, TSG101, and HSP70, together with the chemokine CXCL16 [23].

**Table 2:** The effect of butyrate on cytokine secretion.

Immune cell	Material	Cytokine		Reference
		Up	Down	
Macrophage	C57BL/6 wild‐type mice, Balb/c nude mic, and IBD patients	IL‐13 and CX3CL1	IL‐1β, TNF‐α, CXCL9, and CCL11	[38]
Dendritic cell	Human peripheral blood mononuclear cells	IL‐10	IL‐12 and INF‐γ	[39]
T cell	C57BL/6 mice	IL‐10 and TGF‐β	TNF‐α, IL‐1β, and IL‐17A	[40]
B cell	DBA/1JGpt mice and peripheral blood from RA patients and health volunteers	IL‐10, IL‐13, and TGF‐β	IL‐6, IL‐1β, and TNF‐α	[41]
Natural killer cell	Peripheral blood from health volunteers	—	IFNγ, TNF‐α, IL‐22, sFasL, granzyme A, granzyme B, and perforin	[42]
Neutrophil	C57BL/6J mice and peripheral blood from IBD patients and health volunteers	—	IL‐6, IL‐17A, IL‐22, TNF‐α, IFN‐γ, calprotectin, and LCN2	[43]

Several mechanisms contribute to these anti‐inflammatory effects. One mechanism involves HDAC3 inhibition, which alters p53 acetylation and limits the recruitment of NF‐κB p65 to proinflammatory gene promoters following LPS stimulation [19]. NaBu also attenuates STAT3 phosphorylation, reducing the expression of STAT3‐dependent inflammatory genes [19]. Epigenetic regulation provides an additional layer of control. Butyrate increases histone H3 lysine 9 acetylation (H3K9Ac) at the *NOS2*, *IL6*, and *IL12B* promoter regions while reducing the recruitment of RNA polymerase II (Pol II) and its phosphorylated form (Pol II Ser5P) [44]. As a consequence, the production of nitric oxide (NO), IL‐6, and IL‐12p40 is markedly reduced, but the secretion of TNF‐α and the chemokine MCP‐1 remains largely unaffected [44].

Butyrate also exerts anti‐inflammatory effects through several additional mechanisms. In hepatic macrophages from a mouse model of alcoholic liver disease, oral administration of butyrate reduced the expression of miR‐155 while increasing the levels of SHIP1, SOCS1, and IRAK‐M, all of which are direct targets of miR‐155 in macrophages [45]. These findings suggest that the anti‐inflammatory activity of butyrate may be mediated, at least in part, through restoration of the miR‐155 regulatory network by increasing the expression of SOCS1, SHIP1, and IRAK‐M, although the underlying molecular mechanisms remain to be established. Peroxisome proliferator–activated receptor‐γ (PPAR‐γ) represents another important mediator of butyrate‐induced anti‐inflammatory responses. Pharmacological inhibition of PPAR‐γ with GW9662 abolished the anti‐inflammatory effects of butyrate, whereas butyrate treatment increased the PPAR‐γ expression [46]. These observations suggest that PPAR‐γ is a critical mediator of butyrate‐induced anti‐inflammatory responses. However, the underlying mechanisms remain to be fully elucidated. Iron overload promotes the production of inflammatory cytokines by macrophages and exacerbates colitis, whereas iron chelation alleviates intestinal inflammation. In experimental colitis, oral administration of butyrate disrupted the interaction between Sp1 and HDAC1 at the promoter of *Slc40a1*, the gene encoding ferroportin (FPN), restoring FPN expression that had been suppressed by LPS [38]. This response markedly increased surface FPN expression in macrophages derived from multiple tissues, thereby preserving iron homeostasis during colitis and limiting the production of inflammatory mediators such as TNF‐α.

#### 3.1.5. Effects of Butyrate on Interactions of Macrophage and Other Cells

Once polarized, M2 macrophages orchestrate downstream immune responses through direct cellular interactions and cytokine secretion, representing an important component of butyrate‐mediated immunoregulation. NaBu dose‐dependently inhibits LPS‐induced neutrophil migration, neutrophil extracellular trap (NET) formation, and CXCR6 expression by regulating the release of the exosomal chemokine CXCL16 from LPS‐stimulated RAW264. 7 macrophages [23]. In GPR109a^−^ mice, loss of GPR109a promotes M1 macrophage polarization, resulting in an increased proportion of M1 macrophages within the total macrophage population [32]. This shift is accompanied by an expansion of intestinal Tc1 and Th1 cells, enhanced homing of gut‐derived Tc1 cells to the pancreas, and a reduction in pancreatic regulatory T (Treg) cells [32].

Macrophages also contribute to intestinal barrier maintenance. In M2 macrophages, butyrate activates the WNT–ERK1/2 signaling axis, stimulating the expression of MUC2 (the principal mucin component of the intestinal mucus layer) and SPDEF (a key regulator of goblet cell terminal differentiation) [39]. These changes accelerate epithelial repair while promoting goblet cell differentiation. The resulting reinforcement of the mucus barrier protects epithelial cells from luminal pathogens and other harmful stimuli, creating a favorable microenvironment for colonic epithelial regeneration and differentiation.

### 3.2. Effects of Butyrate on Dendritic Cell (DC)

DCs are specialized antigen‐presenting cells that play a central role in shaping the immune system. During differentiation and maturation, DCs exist in two principal functional states: immature DCs (imDCs) and mature DCs (mDCs), with the immature phenotype predominating in most tissues [39]. Upon exposure to potent immunological stimuli, imDCs undergo maturation, shifting from an antigen‐capturing phenotype to one specialized for antigen presentation and T‐cell activation [39].

#### 3.2.1. Effects of Butyrate on Differentiation of DC

The influence of butyrate on DC differentiation is first reflected in changes in the cellular morphology and phenotype. Compared with the typical mDCs induced by LPS, which display abundant dendritic protrusions and readily form cellular aggregates, butyrate‐treated DCs retain morphological characteristics resembling imDCs: most butyrate‐treated DCs remain in suspension, exhibit a smaller cell size, develop only limited cytoplasmic protrusions, and rarely form cell clusters [39]. These morphological alterations are accompanied by distinct functional changes. In the murine bone marrow–derived DC (BMDC) model, SCFAs, including butyrate, promote actin polymerization by inhibiting HDAC activity and activating the Src family kinase (SFK)/PI3K/Rho GTPase signaling axis, thereby facilitating dendrite extension and enhancing antigen uptake by DCs [47]. These findings indicate that the effects of butyrate on DC morphology cannot be simply interpreted as suppression of immune activation or inhibition of maturation. Instead, while butyrate promotes the acquisition of a tolerogenic phenotype, it simultaneously preserves or even enhances the antigen‐capturing capacity of DCs.

Butyrate also modulates the phenotypic differentiation of DCs. It inhibits the differentiation of conventional DC (cDC) precursors into mature cDCs, resulting in a reduced cDC population. During LPS‐induced maturation, butyrate suppresses HDAC activity and markedly reduces the surface expression of CD40, CD80, CD83, CD86, and major histocompatibility complex class II (MHC II) molecules on DCs, with CD83 expression declining to levels even lower than those observed in imDCs [33, 39, 48–50]. Consistent with their immature phenotype, these butyrate‐treated DCs exhibit a markedly reduced capacity to activate T cells.

#### 3.2.2. Effects of Butyrate on Metabolism of DC

DC function is closely linked to cellular metabolic status, with glycolysis serving as the primary source of energy required for DC activation and cytokine production. Butyrate effectively suppresses this metabolic reprogramming by reducing the total ATP production rate in LPS‐stimulated DCs, primarily through the inhibition of glycolysis‐derived ATP generation [33]. In a murine periodontitis model, oral administration of butyrate downregulated Hif1‐α expression, leading to reduced expression of the rate‐limiting glycolytic enzyme hexokinase 2 (HK2) [33]. This metabolic shift attenuated glycolysis in DCs and ultimately inhibited DC maturation (Table 1). Meanwhile, butyrate markedly decreased the extracellular acidification rate (ECAR), basal mitochondrial oxygen consumption rate (OCR), and spare respiratory capacity, collectively reducing the overall metabolic activity of DCs [49].

#### 3.2.3. Effects of Butyrate on Cytokine of DC

The effects of butyrate on cytokine secretion by DCs are central to its immunomodulatory function and remain one of the most controversial areas of current research. On the one hand, substantial evidence supports the anti‐inflammatory properties of butyrate, demonstrating that it suppresses LPS‐induced production of proinflammatory cytokines, including TNF‐α, IFN‐γ, IL‐1β, IL‐6, and IL‐12, as well as proinflammatory chemokines such as CCL4, CCL5, CXCL9, CXCL10, and CXCL11 by DCs [33, 39, 48] (Table 2). Butyrate also reduces IL‐12 secretion, thereby attenuating the signals required for the priming and activation of naïve CD8^+^ T cells, ultimately limiting CD8^+^ T‐cell activation and IFN‐γ production [51]. Moreover, butyrate induces the expression of IL‐10 and Aldh1a1 in DCs, preferentially promoting the differentiation of naïve T cells into Treg cells while suppressing T helper 17 (Th17) cell development [52].

On the other hand, accumulating evidence has revealed that butyrate may also exert context‐dependent proinflammatory effects. In the murine BMDC model, although oral administration of butyrate inhibited LPS‐induced IL‐12 production by DCs, it simultaneously and markedly increased IL‐23 expression [53]. This effect occurred at the pretranscriptional level and was independent of the Stat3 and ZBP89 signaling pathways. When cocultured with splenocytes, LPS‐stimulated DCs pretreated with butyrate induced higher production of both IL‐17 and IL‐10 [53]. These findings suggest that the effects of butyrate on the cytokine profile of DCs are considerably more complex than a simple anti‐inflammatory response. While butyrate suppresses Th1 immunity by reducing IL‐12 production, it may simultaneously skew immune responses toward a Th17 phenotype through the upregulation of IL‐23. The immunological effects of butyrate are further influenced by the route of administration. In a DSS‐induced colitis model, oral administration of butyrate exacerbated colitis, whereas intraperitoneal administration partially alleviated disease severity [53]. Collectively, these seemingly contradictory findings indicate that the biological effects of butyrate are determined, at least in part, by its site of action, local concentration, and the surrounding tissue microenvironment.

#### 3.2.4. Effects of Butyrate on Interactions of DC and Other Cells

Butyrate regulates DC function primarily through two mechanisms: inhibition of HDAC activity and activation of GPCRs. Early studies mainly emphasized the role of GPR109A signaling. In murine colitis models, activation of GPR109A promoted the anti‐inflammatory phenotype of colonic DCs, induced the differentiation of Treg cells and IL‐10–producing T cells, and stimulated IL‐18 expression in the intestinal epithelium [52]. More recently, study using human monocyte–derived DCs have proposed a more refined mechanistic model. This study demonstrated that butyrate‐induced tolerogenic programming of DCs and the subsequent differentiation of type 1 regulatory T cells (Tr1) require the coordinated actions of HDAC inhibition and GPR109A signaling [49]. Specifically, HDAC inhibition promotes chromatin accessibility at the retinal dehydrogenase 1 (RALDH1) locus, whereas GPR109A signaling initiates RALDH1 transcription [49]. Activation of either pathway alone is insufficient to fully reproduce the immunoregulatory effects of butyrate. RALDH1 catalyzes the conversion of vitamin A into retinoic acid (RA). The resulting RA reinforces RALDH expression through an autocrine mechanism, thereby maintaining the tolerogenic phenotype of DCs [49] (Figure 1). It also acts in a paracrine manner to promote the differentiation of naïve T cells into IL‐10–producing Tr1 cells, which suppress the proliferation of bystander T cells through IL‐10 production [49]. This mechanistic model is further supported by other study showing that butyrate suppresses the expression of proinflammatory genes by inhibiting HDAC activity and remodeling chromatin structure [50].

**Figure 1:**
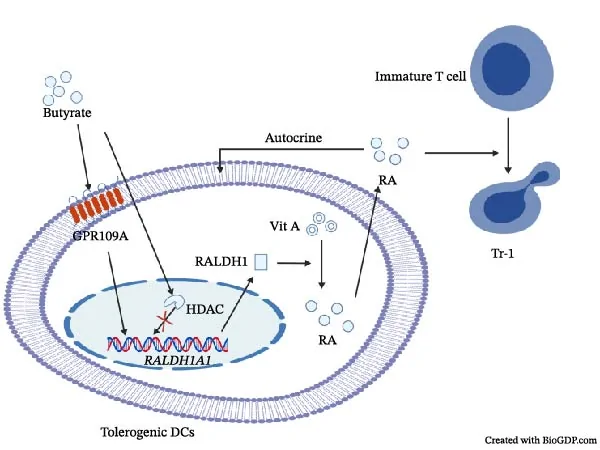
Butyrate induces dendritic cell RA synthesis through dual mechanisms and promotes Tr‐1 cell differentiation. Butyrate simultaneously targets two pathways within dendritic cells. On the one hand, butyrate binds and activates GPR109A on the cell surface, triggering downstream signaling that promotes transcription factor activation specific to ALDH1A1. On the other hand, as an HDAC inhibitor, butyrate enters the nucleus to block HDAC activity, elevating histone acetylation levels and facilitating opening of the chromatin encoding RALDH1, which is ALDH1A1. Activated transcription factors bind to the accessible ALDH1A1 gene promoter region, synergistically initiating and enhancing ALDH1A1 transcription and RALDH1 expression. Highly expressed RALDH1 catalyzes the metabolic conversion of vitamin A to RA. RA acts autocrinely on dendritic cells themselves to maintain their tolerogenic phenotype. Additionally, RA acts paracrinely on naive T cells, directing their differentiation into Tr‐1 cells.

#### 3.2.5. Butyrate‐Mediated Crosstalk Between DCs and Other Immune Cells

The ultimate functional consequences of DC modulation by butyrate are reflected in the regulation of T cells and other immune cell populations. T cells primed by butyrate‐treated DCs exhibited reduced expression of the activation markers CD25 and CD69, decreased IL‐2 secretion, sustained expression of the naïve T‐cell marker CD62L, lower frequencies of Th1 and Th17 cells, and an increased proportion of Foxp3^+^ T cells [33, 49].

Beyond its direct effects on T‐cell differentiation, butyrate also modulates the DC function through additional mediators. Butyrate regulates amphiregulin (AREG) expression in DCs through the GPR43/B lymphocyte–induced maturation protein 1 (Blimp‐1) signaling pathway [54]. As a member of the epidermal growth factor family, AREG promotes epithelial cell proliferation, facilitates tissue repair, and supports Treg cell differentiation [54].

The inhibitory effects of butyrate on DC function, however, may have different implications in the context of antitumor immunity. In preclinical models of melanoma as well as lung and cervical tumors, oral administration of NaBu did not alter the overall abundance of CD11c^+^ cells but reduced the functional activity of APCs by impairing the expression of costimulatory molecules and antigen presentation [55]. Moreover, NaBu impaired cross‐priming, leading to reduced activation and tumor infiltration of ovalbumin (OVA)‐specific CD8^+^ T cells and, consequently, weakened antitumor immune responses [55]. These observations are consistent with the anti‐inflammatory and tolerogenic effects of butyrate reported in other disease models; however, within the tumor microenvironment, such immunoregulatory properties may compromise effective antitumor immunity.

## 4. Effects of Butyrate on Lymphocyte

### 4.1. Effects of Butyrate on T Cell

Regulatory T (Tregs) cells and Th17 cells perform distinct immunological functions through different mechanisms while reciprocally constraining each other’s differentiation and activity. Th17 cells contribute to host defense by producing proinflammatory cytokines, including IL‐17A, IL‐17F, IL‐21, and IL‐22; in contrast, Treg cells suppress excessive antibody production and aberrant immune activation, thereby playing an indispensable role in maintaining immune homeostasis [34].

#### 4.1.1. Effects of Butyrate on Differentiation of T Cell

##### 4.1.1.1. Effects of Butyrate on CD4^+^ T‐Cell Differentiation and Function

Butyrate markedly promotes Treg generation, although this effect is regulated by both its concentration and the presence of T‐cell receptor (TCR) signaling. In a model of anti‐CD3/CD28–stimulated human intestinal lamina propria CD4^+^ T cells (LP CD4^+^ T cells), butyrate neither induced CD4^+^ T‐cell proliferation nor increased the proportion of CD25^+^Foxp3^+^ cells in the absence of TCR stimulation, although it upregulated surface CD25 expression on LP CD4^+^ T cells, an effect that may be associated with HDAC activity [56]. Following TCR stimulation, butyrate reduced CD4^+^ T‐cell proliferation and CD25 expression in a concentration‐dependent manner and markedly suppressed the expansion of all Th subsets, including Th1, Th17, and Th22 cells [56]. Among these subsets, the frequencies of Th1 and Th22 cells declined more prominently at lower butyrate concentrations.

Both natural Treg (nTreg) cells and inducible Treg cells express the lineage‐defining transcription factor forkhead box P3 (Foxp3), and enhanced Treg function is associated with increased histone acetylation at the Foxp3 locus [57]. Butyrate increased the abundance of acetylated histone H3K9 and penta‐acetylated histone H4 at the Foxp3 promoter, enhanced Foxp3 transcription, and promoted the conversion of naïve Foxp3^−^ T cells into Foxp3^+^ Treg cells [57]. Meanwhile, butyrate upregulated the expression of IL‐10 and the transcription factor Helios, leading to an increased proportion of IL‐10–producing Treg cells [57]. Butyrate also promoted the coordinated transcription of Gata3, Gitr, and Sell, whose gene products are essential for Treg activation, function, and migration [57]. Interestingly, butyrate continued to promote the generation of Foxp3^+^ cells even under conditions favoring Th1 or Th17 polarization [58]. When the butyrate concentration was ≤0.25 mM and TGF‐β was present at low concentrations, FOXP3 expression was further increased, accompanied by enhanced differentiation of inducible Treg cells [13].

In addition to Foxp3, butyrate‐treated T cells exhibit elevated expression of several immunoregulatory markers, including inducible T‐cell costimulator (ICOS) and cytotoxic T‐lymphocyte–associated protein 4 (CTLA‐4), both of which enhance the suppressive capacity of Treg cells [59]. Butyrate also suppresses the expression of Th17‐associated transcription factors, including RAR‐related orphan receptor gamma T (RORγt), RA–related orphan receptor α (RORα), Runx1, and Batf [59]. As an endogenous activator of PPAR‐γ, butyrate activates PPAR‐γ signaling, reduces the Th17/Treg ratio, decreases IL‐17 expression, and promotes IL‐10 production, ultimately attenuating inflammatory responses [60]. Moreover, butyrate lowers the Th17/Treg ratio by inhibiting HDAC1 and suppressing IL‐6 and IL‐17 expression as well as the IL‐6/STAT3/IL‐17 signaling pathway while simultaneously enhancing TGF‐β expression [61].

Nevertheless, the effects of butyrate on Th17 cells remain controversial and appear to depend on both its concentration and the differentiation milieu. At low concentrations (0.1–0.25 mM), butyrate suppresses Th17 differentiation in the presence of TGF‐β [13]. By contrast, at higher concentrations (>0.5 mM), NaBu enhances the expression of the Th17 lineage–defining transcription factors RORγt and RORα through the mTOR/S6K signaling pathway [62]. These findings suggest that the immunomodulatory effects of butyrate may involve reshaping the regulatory cytokine milieu rather than simply suppressing a single T‐cell subset.

CTLA‐4–mediated suppressive activity is central to the immunoregulatory function of Treg cells, whereas autophagy contributes to the regulation of CTLA‐4, with mTOR serving as a key mediator of this process [63]. NaBu treatment reduced mTOR expression in Treg cells, inhibited mTOR signaling, and increased the expression of the autophagy marker LC3 [63]. In T cells isolated from patients with acetylcholine receptor–positive myasthenia gravis (AChR‐MG), NaBu markedly increased CTLA‐4 expression on CD4^+^CD25^+^CD127^low^ Treg cells, elevated the proportion of FOXP3^+^CTLA‐4^+^ cells, and significantly enhanced the suppressive activity of Treg cells [63].

##### 4.1.1.2. Effects of Butyrate on CD8^+^ T‐Cell Differentiation and Function

The effects of butyrate on CD8^+^ T cells differ markedly from those observed in CD4^+^ T cells, being characterized predominantly by the potentiation of effector functions rather than the induction of immune tolerance [13, 64–68]. Under cytotoxic T lymphocyte (CTL)–polarizing conditions, NaBu (0.25–1 mM) markedly increased the proportions of IFN‐γ^+^ and granzyme B^+^ CD8^+^ T cells by inhibiting HDAC activity and increasing histone H4 acetylation at the *Tbx21* and *Ifng* promoter regions [64]. However, exposure to 5 mM butyrate induced apoptosis of CTLs [64]. Under Tc17‐polarizing conditions, NaBu promoted the conversion of Tc17 cells toward a CTL‐like phenotype, as evidenced by reduced IL‐17A expression together with increased IFN‐γ and granzyme B production, accompanied by decreased *Rorc* expression and increased expression of *Tbx21* and *Eomes* [64].

Subsequent studies have further demonstrated that NaBu augments the antitumor activity of CD8^+^ T cells through multiple complementary mechanisms. Butyrate directly promoted IFN‐γ production and cytotoxicity of tumor‐infiltrating CD8^+^ T cells through an ID2‐dependent IL‐12 signaling pathway, thereby improving the therapeutic efficacy of oxaliplatin chemotherapy, both in vitro and in vivo [66]. In the tumor‐bearing mice model, butyrate produced by *Roseburia intestinalis* directly bound Toll‐like receptor 5 (TLR5) on CD8^+^ T cells and activated NF‐κB signaling, resulting in increased frequencies of granzyme B^+^, IFN‐γ^+^, and TNF‐α^+^ CD8^+^ T cells and significantly improving the efficacy of anti‐PD‐1 therapy in colorectal cancer (CRC) [69]. Mechanistically, butyrate also increased H3K27 acetylation at the *Cd28* and *Pdcd1* promoter regions, promoting the surface expression of CD28 and PD‐1 on CD8^+^ T cells and further enhancing the therapeutic response to PD‐1 blockade [65]. Moreover, inhibition of class I HDACs together with activation of mTOR signaling enabled butyrate to further potentiate the antitumor activity of CTLs and chimeric antigen receptor (CAR)‐T cells, providing a potential strategy for optimizing adoptive T‐cell therapy [67].

Collectively, the ability of butyrate to enhance CD8^+^ T‐cell effector function contrasts sharply with its well‐established role in promoting Treg differentiation, highlighting the context‐dependent nature of its immunomodulatory activity. In the tumor microenvironment, potentiation of CTL responses is likely to favor antitumor immunity, whereas under autoimmune or chronic inflammatory conditions, the preferential expansion of Treg cells may contribute to the restoration of immune tolerance.

#### 4.1.2. Effects of Butyrate on Metabolism of T Cell

The metabolic effects of butyrate are likewise subset‐specific. In CD4^+^ T cells, butyrate treatment markedly increased both basal and maximal mitochondrial OCR, enhanced OXPHOS in a PPAR‐γ–dependent manner, reduced the glycolytic proton efflux rate (glycoPER) and overall glycolytic activity, and consequently increased the OCR/glycoPER ratio [34, 63]. Butyrate also suppressed glycolysis by downregulating HIF‐1 alpha (HIF‐1α) signaling, thereby promoting Treg differentiation [34] (Table 1). By contrast, NaBu exerts distinct metabolic effects in CD8^+^ T cells. In these cells, butyrate simultaneously enhanced glycolysis and OXPHOS, increased mTOR activity, and promoted CTL effector function [64, 67]. Consistent with these findings, NaBu increased the phosphorylation of mTOR and its downstream target ribosomal protein S6 while elevating the ECAR in CTLs [62]. These distinct metabolic programs may underlie the dual immunomodulatory functions of butyrate, promoting immune tolerance in CD4^+^ T cells while enhancing immune activation in CD8^+^ T cells.

Modulation of key metabolic enzymes can redirect T‐cell differentiation, and fatty acid oxidation is particularly important for inducible Treg generation [70]. In a CD3/CD28‐stimulated murine T‐cell model, intracellular butyrate was directly converted into butyryl‐CoA (BCoA) by acyl‐CoA synthetase short‐chain family member 2 (ACSS2) [70]. BCoA competitively antagonized malonyl‐CoA (MCoA), the physiological inhibitor of carnitine palmitoyltransferase 1A (CPT1A), thereby increasing CPT1A activity, the rate‐limiting enzyme for fatty acid oxidation, and ultimately promoting iTreg generation [70]. Compared with naïve T cells, Th17 cells exhibit a greater uptake of glutamine and glucose [71]. Glutaminase (GLS) and lactate dehydrogenase A (LDHA) are two key metabolic enzymes that regulate glutaminolysis and glycolysis, respectively, and their expression is controlled by the transcription factor c‐Myc [71]. Butyrate inhibited HDAC3, thereby facilitating the acetylation‐dependent degradation of c‐Myc, which reduced the expression of GLS and LDHA and ultimately suppressed Th17 differentiation [71].

#### 4.1.3. Effects of Butyrate on Cytokine of T Cell

In CD4^+^ T cells, butyrate dose‐dependently reduced the production of IL‐1β, IL‐13, IL‐17A, TNF‐α, and IFN‐γ following TCR stimulation; in contrast, in the absence of TCR stimulation, butyrate had no significant effect on IL‐17A or IFN‐γ production [40, 56, 72] (Table 2). Under Th1‐polarizing conditions, butyrate enhanced IFN‐γ production, whereas under Th17‐polarizing conditions, it suppressed IL‐17 production [59]. Despite these context‐dependent effects, butyrate consistently increased IL‐10 production in T cells under both conditions [59]. Blimp‐1, a key regulator of IL‐10 expression in T cells, was expressed at higher levels in butyrate‐treated CD4^+^ T cells [59].

Aryl hydrocarbon receptor (AhR) directly binds to the IL‐22 promoter, whereas HIF‐1α, a transcription factor activated by hypoxia, has also been shown to regulate IL‐22 production by CD4^+^ T cells [73]. Butyrate promoted the expression and activity of HIF‐1α and AhR through GPR41, enabling these two transcription factors to cooperatively enhance IL‐22 production [73]. Moreover, butyrate inhibited HDAC activity, reduced trimethylation at the hypoxia response element (HRE) within the IL‐22 promoter, and enhanced HIF‐1α binding to this regulatory region, thereby further increasing IL‐22 expression [73]. STAT3 and mTOR signaling also participate in the regulation of HIF‐1α and AhR expression. Butyrate enhanced STAT3 activation, which subsequently activated the mTOR pathway and promoted IL‐22 production [73]. Interestingly, STAT3 and mTOR differentially regulated HIF‐1α and AhR expression: Inhibition of STAT3 suppressed the expression of both HIF‐1α and AhR, whereas inhibition of mTOR selectively reduced AhR expression without affecting HIF‐1α [73].

In CD8^+^ T cells, butyrate enhances the production of IFN‐γ, TNF‐α, and granzyme B through multiple mechanisms. First, NaBu promotes the expression of IFN‐γ and granzyme B in CTLs independently of GPR41‐ and GPR43‐mediated signaling through HDAC inhibition [64]. Further studies have shown that NaBu enhances mTOR activity, a central regulator of cellular metabolism, while simultaneously inhibiting class I HDACs, thereby further increasing the production of effector molecules such as IFN‐γ and TNF‐α [67]. This effect is further potentiated in the presence of IL‐12 [66]. Second, butyrate enhances the production of antitumor cytokines by CTLs, including IFN‐γ, TNF‐α, and IL‐2, through a TCR‐dependent signaling pathway [65]. Mechanistically, butyrate initiates a cascade of protein phosphorylation events that promotes Ca^2+^ release from endoplasmic reticulum stores, ultimately augmenting cytokine secretion by CTLs [65].

Collectively, current evidence indicates that the immunomodulatory effects of butyrate on T cells cannot be simply described as promoting Treg cells while suppressing effector T cells. Instead, its effects are governed by a complex interplay between the butyrate concentration and the surrounding microenvironment. With respect to concentration, low levels of NaBu (≤0.25 mM) preferentially promote Treg differentiation [13]. Under CD3 stimulation, intermediate concentrations (>0.25 mM) induce the expression of effector molecules such as IFN‐γ, whereas high concentrations (>5 mM) may trigger T‐cell apoptosis [64]. This concentration‐dependent response suggests that NaBu may exert distinct immunological functions at different anatomical sites, such as the high‐butyrate environment of the intestinal lumen versus the relatively low‐butyrate environment of the peripheral circulation. The source of butyrate also influences its biological effects. Endogenously produced butyrate derived from the gut microbiota and exogenously administered butyrate exhibit distinct tissue distribution patterns and biological activities. Orally administered butyrate primarily acts within the intestinal tract, whereas intraperitoneal administration bypasses intestinal first‐pass metabolism and directly targets peripheral immune cells. These findings highlight that discrepancies among studies may arise not only from differences in butyrate concentration but also from variations in tissue distribution, route of administration, and the local immune microenvironment.

### 4.2. Effects of Butyrate on B Cell

Extensive evidence indicates that butyrate directly modulates B‐cell metabolism, lineage commitment, and anti‐inflammatory cytokine production, thereby playing a pivotal role in autoimmune diseases. B cells are central components of the adaptive immune system and contribute to the maintenance of immune homeostasis through multiple mechanisms. By coordinately regulating multiple aspects of B‐cell function, butyrate promotes the transition from a proinflammatory effector state to a tolerogenic phenotype, thereby establishing both local and systemic immunosuppressive networks.

#### 4.2.1. Effects of Butyrate on Differentiation of B Cell

##### 4.2.1.1. Suppression of Plasma Cell Differentiation by Butyrate

Butyrate suppresses plasma cell differentiation through a dose‐dependent mechanism. Mechanistically, NaBu inhibits histone deacetylation at the host genes of microRNAs targeting Aicda and Prdm1 mRNAs, thereby increasing the expression of these microRNAs and consequently reducing the expression of activation‐induced cytidine deaminase (AID) and Blimp‐1 [74]. AID is indispensable for both class‐switch recombination (CSR) and somatic hypermutation (SHM), which are essential for generating protective antibodies against microbial pathogens as well as IgE‐mediated allergic responses and pathogenic autoantibodies in autoimmune diseases [74]. Blimp‐1 is a master regulator of plasma cell differentiation and, together with AID, is required for the generation of class‐switched, affinity‐matured antibodies and autoreactive antibody responses [75]. Accordingly, by downregulating AID and Blimp‐1, NaBu dose‐dependently suppresses CSR to IgG, IgA, and IgE; reduces the frequencies of surface IgG^+^ and IgA^+^ B cells; decreases the secretion of IgG and IgA; and inhibits human plasma cell differentiation without affecting B‐cell viability or proliferation [74]. Because Xbp1 is transcriptionally regulated by Blimp‐1 and is required for immunoglobulin secretion by plasma cells, reduced Blimp‐1 expression also leads to decreased Xbp1 expression [75] (Figure 2). In lupus‐prone mice, oral administration of NaBu suppresses the expression of AID and Blimp‐1, inhibits plasma cell differentiation, and reduces the production of class‐switched autoantibodies [74]. Consistently, serum titers of IgG1 and IgG2a autoantibodies against dsDNA, RNP/Sm, RNA, histones, and nuclear antigens are markedly reduced [74]. These changes are accompanied by reduced numbers of B cells and plasma cells, near‐complete resolution of cutaneous lesions, and the absence of detectable renal IgG1/IgG2a deposition or glomerular injury, suggesting that modulation of butyrate‐related pathways may represent a potential therapeutic avenue in lupus [74]. Furthermore, the reduced affinity of IgM antibodies suggests that NaBu may also have therapeutic potential in allergic diseases [74].

**Figure 2:**
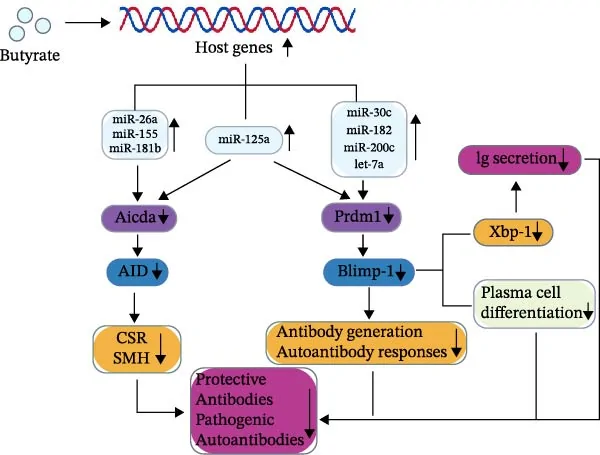
Butyrate epigenetically upregulates miRNAs targeting AID and Blimp‐1, leading to suppression of antibody and autoantibody responses. Butyrate acts as a dose‐dependent histone deacetylase (HDAC) inhibitor within host cells. This inhibition leads to histone acetylation and upregulates miRNAs that target Aicda and Prdm1 mRNAs (miR‐26a, miR‐155, and miR‐181b for Aicda; miR‐30c, miR‐182, miR‐200c, and let‐7a for Prdm1; and miR‐125a for both). The upregulated miRNAs targeting Aicda and Prdm1 mRNAs lead to their degradation and results in significant downregulation of AID and Blimp‐1. Reduced AID protein level impairs CSR and SHM. Reduced Blimp‐1 protein level impairs plasma cell differentiation; deregulates Xbp‐1, a key transcription factor promoting secretion in plasma cells; and decreases the generation of Ab and autoantibody responses. The butyrate‐induced, miRNA‐mediated suppression of AID and Blimp‐1 culminates in a reduction of both protective antibodies and pathogenic autoantibodies.

##### 4.2.1.2. Promotion of Regulatory B‐Cell Differentiation by Butyrate

However, the effects of butyrate on B‐cell differentiation are not uniformly suppressive. Immature B cells differentiate into B1 cells and transitional B cells, the latter of which subsequently give rise to regulatory B cells (Bregs), follicular B cells, and marginal zone B cells, and the relative abundance of these subsets is closely associated with the magnitude of immune responses [35]. During B‐cell differentiation, butyrate reshapes lineage commitment by modulating key signaling pathways, suppressing differentiation toward conventional plasma cells or memory B cells while favoring the generation of Bregs [35]. In mice treated with butyrate, the proportion of peripheral Bregs is positively correlated with butyrate levels, accompanied by an expansion of Bregs and a reduction in transitional and follicular B cells [35]. In a collagen‐induced arthritis (CIA) model, oral administration of butyrate suppresses the differentiation of immature B cells into transitional B cells through FFA2 signaling while promoting the differentiation of transitional B cells into Bregs and limiting their differentiation into follicular B cells [35].

Mitochondrial respiration also influences plasma cell differentiation, and reduced mitochondrial activity together with diminished superoxide production appears to favor this process. Consistent with this notion, butyrate decreases mitochondrial superoxide levels. In an OVA/complete Freund’s adjuvant (CFA)–induced mouse model, the ability of butyrate to induce plasma cell differentiation was abolished following treatment with the mitochondrial superoxide inducer phorbol 12‐myristate 13‐acetate (PMA) or the mitochondrial ROS inducer 2‐DG, suggesting that suppression of mitochondrial superoxide is required for butyrate‐induced plasma cell differentiation [76]. Notably, the CD138^high^ plasma cells induced under these conditions are considered immunoregulatory rather than pathogenic, owing to their production of IL‐10, TGF‐β, IL‐35, and highly glycosylated IgG antibodies [76]. Butyrate dose‐dependently promotes the differentiation of naïve B cells into CD138^high^ plasma cells by inhibiting HDAC3 while simultaneously enhancing the expression of anti‐inflammatory cytokines [76]. This process is likely mediated through transcriptional reprogramming of plasma cell–associated genes, including the upregulation of Prdm1 and Irf4 together with the suppression of Irf8 and Aicda [76].

More importantly, accumulating evidence suggests that, under specific pathological conditions, butyrate can reverse its inhibitory effects and instead promote the generation of effector plasma cells. In a mouse model of vancomycin‐induced gut microbiota depletion‐associated humoral immunosuppression, oral administration of butyrate markedly upregulated Blimp‐1 expression by activating the Akt–mTOR pathway, thereby promoting the generation of CD138^+^ plasma cells and alleviating the impaired humoral immune response to rabies vaccination caused by vancomycin treatment [77]. This finding contrasts sharply with the previously described inhibitory effect of high‐dose butyrate on plasma cell differentiation. One possible explanation is that, within the antibiotic‐disrupted “deficient” microenvironment, butyrate primarily serves as an energy substrate to support intestinal cellular metabolism, resulting in relatively low circulating butyrate concentrations that favor the generation of effector plasma cells.

#### 4.2.2. Effects of Butyrate on Metabolism of B Cell

At the metabolic level, butyrate directly influences B‐cell activation and function by reprogramming cellular energy metabolism. Following β‐oxidation, NaBu is converted into acetyl‐CoA, which enters the tricarboxylic acid (TCA) cycle and serves as a metabolic substrate to enhance OXPHOS, glycolysis, and fatty acid synthesis [74]. Because B‐cell differentiation into PCs requires fatty acid synthesis, B cells utilize butyrate‐derived acetyl‐CoA, which is subsequently converted into MCoA to promote fatty acid synthesis and facilitate PC differentiation [78]. Evidence from a vancomycin‐treated mouse model further demonstrated that oral administration of butyrate significantly increased mitochondrial membrane potential, mitochondrial complex I activity, and intracellular ATP levels while enhancing the phosphorylation of Akt and mTOR [77]. In contrast, inhibition of the Akt–mTOR pathway completely abolished butyrate‐induced Prdm1 upregulation and PC generation [77].

#### 4.2.3. Effects of Butyrate on Cytokine of B Cell

Cytokine secretion represents one of the central mechanisms underlying the immunoregulatory properties of B cells. Following butyrate treatment, the levels of IL‐6, IL‐1β, IL‐17, IL‐21, and TNF‐α were reduced in mice, whereas the levels of IL‐10, IL‐13, and TGF‐β were increased, resulting in decreased production of proinflammatory cytokines and enhanced production of anti‐inflammatory cytokines, thereby effectively maintaining immune tolerance [35, 41] (Table 2). Butyrate increases the expression of RORα, which directly binds to the IL‐10 promoter and consequently expands the population of IL‐10–producing B cells [41]. In a murine model of Sjögren’s syndrome, intraperitoneal injection of butyrate reduced the expression of NR1D1 in the salivary glands, accompanied by increased expression of NFIL3, which is negatively regulated by NR1D1, thereby suppressing IL‐17–producing B cells and reducing the production of proinflammatory cytokines [41]. Butyrate also indirectly regulates B‐cell physiology through the modulation of the gut microbiota. In a murine model of antigen‐induced arthritis (AIA), oral NaBu supplementation altered the composition of the gut microbiota [79]. These microbial alterations reshaped the profile of tryptophan‐derived metabolites, with a marked increase in 5‐hydroxyindoleacetic acid (5‐HIAA), the major metabolite of serotonin [79]. 5‐HIAA activated the AhR in B cells, increased the expression of Cyp1a1 and IL‐10, supported Breg function, and simultaneously suppressed the differentiation of germinal center (GC) B cells and plasmablasts, ultimately attenuating arthritis progression [79] (Table 1).

### 4.3. Effects of Butyrate on Natural Killer Cell (NK Cell)

Natural killer (NK) cells play essential roles not only in antitumor immunity and antiviral defense but also in the coordination of inflammatory responses. By secreting proinflammatory cytokines, NK cells activate and polarize other immune cell populations, thereby serving as a bridge between innate and adaptive immunity. Based on the differential expression of CD56, human NK cells are broadly classified into two subsets: CD56^bright^ and CD56^dim^ cells. CD56^dim^ cells account for ~90% of peripheral blood NK cells and exhibit potent cytotoxic activity, whereas CD56^bright^ cells represent ~10% of the circulating NK‐cell population; express high levels of cytokine receptors, including IL‐2R, IL‐12R, IL‐15R, and IL‐18R; and produce large amounts of cytokines, particularly IFN‐γ [80].

#### 4.3.1. Effects of Butyrate on Differentiation and Cytokine of NK Cell

Butyrate modulates the NK cell function by regulating the expression of surface receptors. In human NK cells stimulated with IL‐12 and IL‐15, these cytokines upregulated the expression of TNF‐related apoptosis–inducing ligand (TRAIL), NKp44, NKp30, and NK receptors, whereas butyrate suppressed the responsiveness of both CD56^light^ and CD56^dim^ NK‐cell subsets to IL‐12/IL‐15 stimulation [80]. CCD71 (transferrin receptor) is a marker of NK‐cell activation and an indirect indicator of metabolic activity, and its upregulation correlates positively with enhanced glycolysis and cellular activation under the control of the mTORC1 signaling pathway [80]. Resting NK cells express only low levels of CD71, whereas butyrate treatment consistently and markedly reduced IL‐12/IL‐15–induced CD71 expression in both NK‐cell subsets [80]. With respect to direct cytotoxicity, butyrate dose‐dependently reduced the surface expression of NKG2D and NKp46, suggesting that nuclear translocation of NF‐κB p50 was almost completely inhibited [42]. The reduced expression of NKG2D and NKp46 was accompanied by impaired NKG2D‐ and NKp46‐dependent cytotoxicity [42]. When NK cells were cocultured with K562 target cells, butyrate decreased the proportion of Annexin V–positive K562 cells, indicating attenuated NK cell–mediated cytotoxicity [42]. Butyrate also acts through GPR109A in Kupffer cells and hepatocytes to stimulate IL‐18 production, which promotes the maturation of liver‐resident NK (LrNK) cells by improving mitochondrial OXPHOS via IL‐18R signaling [81].

Beyond receptor‐mediated regulation, the production of cytotoxic molecules and cytokines by NK cells is tightly controlled by receptor‐dependent signaling. Butyrate markedly reduced the production of IFN‐γ, TNF‐α, IL‐22, soluble Fas ligand (sFasL), granzyme A, granzyme B, and perforin [42, 80] (Table 2). The reduction in IFN‐γ secretion may be attributable, at least in part, to the butyrate‐mediated suppression of CD25 expression.

#### 4.3.2. Effects of Butyrate on Metabolism of NK Cell

NK‐cell function depends on energy generated through glycolysis and oxidative metabolism. Peripheral blood NK cells isolated from patients with IBD exhibit metabolic dysregulation, mitochondrial defects, and impaired cytotoxic activity [80]. NK‐cell responses to cytokine stimulation require adequate metabolic support, with the metabolic demands of activation being governed by mTORC1‐, c‐Myc‐, and HIF‐1α‐dependent signaling pathways. In human NK cells stimulated with IL‐12 and IL‐15, butyrate suppressed mTORC1 activity in both CD56^light^ and CD56^dim^ NK‐cell subsets, reduced c‐Myc expression to basal levels, and markedly attenuated the cytokine‐induced increases in mitochondrial mass and membrane potential [80]. Cytokine stimulation enhanced both glycolysis and OXPHOS in NK cells and was accompanied by a marked increase in HK2 expression, whereas butyrate significantly inhibited cytokine‐induced HK2 upregulation [80]. Collectively, the inhibitory effects of butyrate appear to be mediated primarily through the suppression of HK2, leading to impaired mitochondrial function, reduced glycolysis and OXPHOS, and ultimately compromised NK‐cell activity [80].

#### 4.3.3. Other Effects of Butyrate on NK Cell

Unlike its direct inhibitory effects on peripheral blood NK cells described above, butyrate can exert opposing immunomodulatory effects through indirect mechanisms under specific tissue microenvironments or pathological conditions. Butyrate can also regulate NK‐cell function indirectly by modulating other immune cell populations. Cytosine‐phosphate‐guanine (CpG)–stimulated eosinophils produce the proinflammatory cytokine IL‐12 and the chemokine CXCL10, both of which are essential for NK‐cell recruitment and activation in vivo [82]. Treatment of CpG‐stimulated eosinophils with butyrate reduced the expression and production of CXCL10 and IL‐12, thereby diminishing their capacity to recruit NK cells and induce IFN‐γ production [82]. In a mouse model of hepatocellular carcinoma (HCC), oral administration of butyrate markedly increased CXCL11 expression within tumor tissues, thereby promoting NK‐cell recruitment into the tumor microenvironment and enhancing local antitumor immunity [83]. In a CRC liver metastasis (CLM) model, oral administration of NaBu attenuated pathogen‐induced depletion of hepatic NKT cells and consequently partially suppressed CLM progression [84].

Collectively, the immunoregulatory effects of butyrate on NK cells are highly dependent on the tissue microenvironment (e.g., peripheral blood versus liver tumors) and the cellular target through which it acts (direct versus indirect regulation).

## 5. Effects of Butyrate on Neutrophil

Neutrophils are innate immune cells that respond rapidly to invading pathogens and coordinate the recruitment and activation of other immune cell populations through the production of cytokines. As the first responders at sites of inflammation, neutrophils play a pivotal role in protecting the host against bacterial infections. Microbiota‐derived butyrate modulates multiple aspects of neutrophil activity, including activation, migration, and the production of inflammatory mediators. By regulating the secretion of proinflammatory mediators and limiting NET formation, butyrate also exerts broad immunomodulatory effects on neutrophils.

### 5.1. Effects of Butyrate on Secretion of Neutrophil

Activated neutrophils recruit and activate monocytes, macrophages, and other adaptive immune cells through the release of a broad spectrum of chemokines, thereby sustaining inflammatory responses [85]. High‐mobility group box 1 (HMGB1) contributes to the pathogenesis of a wide range of infectious and sterile inflammatory disorders [43]. NF‐κB is a key transcription factor that drives the expression of numerous genes [43]. In an LPS‐induced mouse model of acute lung injury (ALI), the expression of NF‐κB and HMGB1 was markedly elevated, whereas intraperitoneal injection of NaBu significantly suppressed both their expression and release [43]. Similarly, in a mouse model of acute respiratory distress syndrome (ARDS), intraperitoneal injection of butyrate treatment significantly reduced neutrophil infiltration and markedly decreased both the transcription and protein expression of IL‐8, IL‐6, and TNF‐α, which are critical mediators of neutrophil recruitment and activation [86].

Numerous studies have demonstrated that butyrate treatment markedly downregulates the mRNA expression of proinflammatory cytokines, including IL‐6, TNF‐α, and IFN‐γ, in LPS‐stimulated neutrophils, while simultaneously reducing the release of calprotectin, LCN2, CINC‐2αβ, and multiple chemokines [43, 85, 87, 88] (Table 2). However, one study reported increased CINC‐2αβ release following butyrate treatment using the air‐pouch assay, which may be attributable to the sterile inflammation model employed in that study [89]. In the air‐pouch model and in vitro chemotaxis assays, butyrate significantly enhanced neutrophil recruitment to inflammatory sites, an effect associated with the upregulation of L‐selectin expression on neutrophils and increased release of the chemokine CINC‐2αβ [89]. However, this promigratory effect was primarily observed in acute local injection models, whereas prolonged systemic administration of butyrate reduced neutrophil infiltration into the peritoneal cavity. These findings suggest that the regulatory effects of butyrate on neutrophil migration may be temporally dependent, promoting early neutrophil recruitment to facilitate pathogen clearance while limiting excessive infiltration during later stages to minimize tissue injury.

### 5.2. Effects of Butyrate on NETs of Neutrophil

NETs constitute a critical host defense mechanism against invading pathogens. During the specialized process of NETosis, neutrophils release extracellular reticular structures composed of histones, granule proteins, and decondensed genomic DNA, collectively referred to as NETs [90]. NETs play an essential role in host defense. However, accumulating evidence indicates that excessive or sustained NET release contributes to the pathogenesis of a variety of inflammatory and autoimmune diseases, including systemic lupus erythematosus (SLE), vasculitis, and IBD [85]. In a mouse model of abdominal aortic aneurysm (AAA), oral administration of butyrate markedly reduced the expression of NOX2 in neutrophils stimulated with LPS or TNF‐α, thereby suppressing inflammation‐induced NET formation [90]. Autophagy is also required for NET release. In a *S. aureus*–induced bovine neutrophil model, NaBu inhibited autophagy by activating the mTOR and AKT signaling pathways, thereby reducing NET release and significantly decreasing the release of the major NET components, including myeloperoxidase (MPO), histones, and neutrophil elastase (NE) [91]. Excessive production and release of MPO and ROS have been shown to directly contribute to tissue injury, and NaBu effectively suppresses MPO production by neutrophils [85]. Mechanistically, butyrate may reduce PMA‐induced phosphorylation of p47phox by activating GPR43 and Gi/o proteins, thereby attenuating superoxide production [92]. Alternatively, NaBu may suppress the expression of Nox2, Cyba, Ncf1, and Ncf4 in neutrophils, thereby reducing the abundance of oxygen‐dependent NADPH oxidase complex components required for ROS generation and ultimately limiting ROS production [93].

### 5.3. Other Effects of Butyrate on Neutrophil

Reduced neutrophil apoptosis has also been implicated in the development of systemic inflammatory response syndrome and multiple organ dysfunction syndrome. Butyrate induces apoptosis in both resting and activated neutrophils by upregulating bax mRNA expression while suppressing the expression of mcl‐1 and a1 mRNA [94].

## 6. Conclusion and Prospect

The relationship between the gut microbiota and human health has attracted increasing attention in recent years. However, the composition of the gut microbiota is profoundly influenced by multiple factors, including dietary habits, geographic distribution, and ethnicity, making it difficult to establish a unified model. Consequently, microbiota‐derived metabolites have emerged as a major focus of current research. This review systematically summarizes the immunomodulatory effects of butyrate on macrophages, DCs, T cells, B cells, NK cells, and neutrophils from multiple perspectives, including metabolic reprogramming, cell proliferation and differentiation, cytokine regulation, and cellular function. A central theme throughout this review is that the immunological effects of butyrate are neither intrinsic nor unidirectional but are highly dependent on its concentration, target cell type, tissue microenvironment, and pathophysiological state. This context dependence may explain why butyrate promotes immune tolerance in autoimmune diseases, enhances the cytotoxicity of CD8^+^ T cells in the tumor microenvironment, and promotes the bactericidal activity of macrophages during infection. Nevertheless, several important questions remain to be addressed. Why does butyrate exert opposing immunomodulatory effects at different concentrations, and what are the key molecular switches that govern these concentration‐dependent responses? How do HDAC inhibition and GPCR signaling cooperate or compete in mediating the immunoregulatory effects of butyrate across different immune cell types, and is their relative contribution determined by cellular context? Are the discrepant effects of butyrate observed in vitro and in vivo, as well as across different routes of administration, driven by differences in local concentration gradients and metabolic kinetics within the tissue microenvironment? Furthermore, the causal relationship between the depletion of butyrate‐producing bacteria and the progression of autoimmune diseases, as well as the extent to which findings from animal models can be translated into human disease, remains to be established.

Addressing these questions will deepen our understanding of the mechanisms by which butyrate regulates immune responses and provide important insights into the development of butyrate‐based therapeutic strategies for immune‐related diseases. Despite its therapeutic potential, the clinical application of butyrate for autoimmune and inflammatory diseases remains subject to substantial challenges. Most of the available evidence has been derived from in vitro cellular studies and animal models, whereas high‐quality clinical evidence remains limited. The low oral bioavailability of butyrate, its rapid metabolism in vivo, and the difficulty in maintaining therapeutically relevant concentrations, together with its potentially opposing immunomodulatory effects in cancer and autoimmune diseases, represent major barriers to clinical translation. Future studies should focus on the development of targeted delivery systems, the identification of more stable butyrate analogs, and the precise indirect modulation of the butyrate‐producing gut microbiota. Overall, this review aims to provide a conceptual framework for understanding the complex immunomodulatory effects of butyrate rather than advocating its immediate use as an established therapeutic intervention.

## Author Contributions

Juzhong Zhang, Shuyan Shen, and Jiamin Cao collected information. Juzhong Zhang, Yangbo Zhou, and Yunping Li classified the data. Juzhong Zhang, Boding Tong, and Yuxian Shi wrote the paper. Xin Qi had primary responsibility for final content.

## Funding

This study was supported by the National Nature Science Foundation of China (Grant 82301264, Xin Qi) and the Science and Technology Innovation Program of Hunan Province (Grant 2023RC3084, Xin Qi).

## Disclosure

All authors have read and approved the final manuscript.

## Conflicts of Interest

The authors declare no conflicts of interest.

## Data Availability

Data described in the manuscript, code book, and analytic code will be made publicly and freely available without restriction at PubMed.
